# Broadening the phenotype of the *TWNK* gene associated Perrault syndrome

**DOI:** 10.1186/s12881-019-0934-4

**Published:** 2019-12-18

**Authors:** Bálint Fekete, Klára Pentelényi, Gabor Rudas, Anikó Gál, Zoltán Grosz, Anett Illés, Jimoh Idris, Gabor Csukly, Andor Domonkos, Maria Judit Molnar

**Affiliations:** 1 Institute of Genomic Medicine and Rare Disorders, Semmelweis University, 26 Üllői Rd, Budapest, 1085 Hungary; 2 MR Research Centre, Semmelweis University, Budapest, Hungary; 3 Department of Psychiatry and Psychotherapy, Semmelweis University, Budapest, Hungary; 4 Institute of Experimental Medicine of the Hungarian Academy of Sciences, Budapest, Hungary

**Keywords:** Perrault syndrome, *TWNK*, Hyperintense cerebellar signal, Spastic ataxia

## Abstract

**Background:**

Perrault syndrome is a genetically heterogenous, very rare disease, characterized clinically by sensorineural hearing loss, ovarian dysfunction and neurological symptoms. We present the case of a 33 years old female patient with *TWNK*-associated Perrault syndrome. The *TWNK* gene is coding the mitochondrial protein Twinkle and currently there are only two reports characterizing the phenotype of *TWNK*-associated Perrault syndrome. None of these publications reported about special brain MRI alterations and neuropathological changes in the muscle and peripheral nerves.

**Case presentation:**

Our patients with *TWNK*-dependent Perrault syndrome had severe bilateral hypoacusis, severe ataxia, polyneuropathy, lower limb spastic paraparesis with pyramidal signs, and gonadal dysgenesis. Psychiatric symptoms such as depression and paranoia were present as well. Brain MRI observed progressive cerebellar hyperintensive signs associated with cerebellar, medulla oblongata and cervical spinal cord atrophy. Light microscopy of the muscle biopsy detected severe neurogenic lesions. COX staining was centrally reduced in many muscle fibers. Both muscle and sural nerve electron microscopy detected slightly enlarged mitochondria with abnormal cristae surrounded by lipid vacuoles. In the sural nerve, dystrophic axons had focally uncompacted myelin lamellae present. Genetic investigation revealed multiple mtDNA deletion and compound heterozygous mutations of the *TWNK* gene (c.1196 A > G, c.1358 G > A).

**Conclusion:**

This study demonstrates that *TWNK* associated Perrault syndrome has a much broader phenotype as originally published. The coexistence of severe hypoacusis, spastic limb weakness, ataxia, polyneuropathy, gonadal dysgensia, hyperintense signals in the cerebellum and the presence of the mtDNA multiple deletion could indicate the impairment of the *TWNK* gene. This is the first report about pyramidal tract involvement and cerebellar MRI alteration associated with *TWNK*-related Perrault syndrome.

## Background

TWNK (previously C10orf2) is a gene (chromosome 10) encoding the Twinkle protein, an adenine nucleotide-dependent DNA helicase acting in the mitochondria, with a focal function in the maintenance of the mtDNA integrity [15, 11]. Mutations of *TWNK* are well-described in mitochondrial DNA depletion syndrome 7 (MTDPS7) which is also known as infantile-onset spinocerebellar ataxia (infantile onset-SCA) [13] and autosomal dominant progressive external ophthalmoplegia 3 (autosomal dominant-PEO3) [6]. In 2014, a new phenotype Perrault syndrome was associated to *TWNK* gene alterations [12]. The observations of Morino et al. were supported by Demain [3] and Oldak [14] and now, by our study as well.

Perrault syndrome is a very rare disease (< 1/1.000.000), characterized by sensorineural hearing loss, ataxia and ovarian dysfunction. Additional features can include a variety of neurologic symptoms, such as nystagmus, dysarthria, and muscle weakness. In other Twinkle-related syndromes external ophthalmoplegia, migraine, epilepsy, cardiomyopathy, cataracts, and psychiatric symptoms could further complicate the issue as well [6, 7, 10].

The brain MRI scans of patients with Twinkle mutations show a very diverse landscape. In ad-PEO3, mild cortical atrophy and white matter abnormalities in subcortical and periventricular regions were described [14]. In mitochondrial DNA depletion syndrome 7, stroke-like lesions and edematous lesions were described with no predilection, and in other cases, cerebellar atrophy – sometimes together with cerebellar white matter lesions – were reported [4, 6]. In the *TWNK*-related Perrault syndrome, cerebellar atrophy is the consistent MRI finding [3]. In *LARS2*- and *HARS2*-associated Perrault syndrome, no data were published about the brain imaging, while in *CLPP*-associated Perrault syndrome, high signal intensity in the corticospinal tract deep white matter could be found [8]. In *HSD17B4*-dependent Perrault syndrome and in *TWNK*-dependent Perrault syndrome, cerebellar atrophy was only described besides non-specific findings [9].

In Perrault syndrome, besides the central nervous system (CNS) symptoms, sensorimotor peripheral neuropathy is an important and frequent finding. Both axonal and demyelinating forms of neuropathies have been described by NCS (nerve conduction studies) [12]. We have one report about type II myofiber atrophy [12] but none of the published studies investigated the neuropathological alterations of the muscle and peripheral nerves in details.

The phenotypic heterogeneity of Perrault syndrome is accompanied with genetic heterogeneity. The most commonly affected genes are *HARS2, HSD17B4, LARS2, CLPP, ERAL1* [2, 3] and more authors recently proposed *SGO2* and *CLDN14* [5]. Out of the six currently known genes, *HSD17B4* is involved in peroxisomal fatty acid beta-oxidation, *LARS2*, *HARS2, CLPP*, and the newly associated *TWNK* and *ERAL1* play a role in mitochondrial function. *TWNK* mutation may result in mtDNA depletion in case of mitochondrial DNA depletion syndrome 7, or in mtDNA multiple deletions in Perrault syndrome and autosomal dominant external ophthalmoplegia 3 [6].

Here we aim to report the detailed phenotypic (clinical, laboratory, imaging and neuropathological) characteristics and the genetic data of our case with Perrault syndrome caused by *TWNK* mutations.

## Case presentation

A Hungarian patient and her family are the subject of this report. Written informed consent was obtained from all persons involved in the investigations. The patient gave informed, written consent for publication of the history and examinations. This article is a retrospective case study; it has the approval of the Ethical Committee of the Medical Research Council (TUKEB 44599–2/2013/EKU 535/2013).

Detailed routine neurological and psychiatric examinations were performed, and family history was taken. Symptom Checklist-90-Revised (SCL-90-R) test was performed to measure the patient’s scores on 9 primary symptom dimensions (somatization, hostility, depression, obsession-compulsion, interpersonal sensitivity, anxiety, phobic anxiety, paranoid ideation and psychosis) and also, on an additional subscale, the presence of sleep and memory problems, which altogether provide the global severity index (GSI). In the process of analysis, the SAS system was used (Release 9.1 TS Level 1 M3, Statistical Analysis System, SAS-Institute USA). The nerve conduction study was performed using standard methods (Dantech Keypoint, Denmark), the median, ulnar motor and sensory nerves, peroneal and tibial motor nerves, and sural nerve were investigated. The patient was examined by MRI in 2008 and 2016 (3 Tesla, Philips Achieva) in the MR Research Center of Semmelweis University. FLAIR, MPR, T1 and T2 weighted scans were made, analyzed and compared. The muscle and nerve histology and histochemistry were assessed by standard methods. Transversely oriented muscle blocks were obtained from the quadriceps femoris muscle. Cryostat sections were obtained from both the muscle and sural nerve specimen for light microscopy, and for glutaraldehyde-fixed and Epon-embedded material for electron microscopic studies.

DNA was isolated from both blood and muscle. The mtDNA deletion was detected by long range PCR. The sequence analysis for *POLG1* gene was performed by Sanger sequencing. The sequence was compered to human reference genome (hg38) by NCBI Blast software. *HSD17D4* and *HARS2* genes were analyzed at Centogene. The NGS panel was developed in the Institute of Genomic Medicine and Rare Disorders, the panel consisted of 51 genes which were previously described to play a role the mtDNA maintenance. Library preparation was performed with SureSelect QXT kit (Agilent Technologies, CA, USA) according to the manufacturer’s instructions. Panel: 98191 kbp region size, 1358 probes. Sequencing: MiSeq reagent kit v2, 300 cycles, Illumina.MiSeq platform (Illumina, San Diego, USA) was used for the NGS run. Analysis and variant calling was performed with SureCall software (Agilent Technologies, CA, USA). We filtered for known disease-causing/benign variants (HGMD, Alamut, dbSNP, ensembl.org, PubMed, RGD, ftp.expasy.org, DMDM databases) and for rare variants (minor allele frequency < 0.5% in Exome Aggregation Consortium (ExAC) and 1000Genomes (1000G), Genome Aggregation Database (genomeAD) and Trans-Omics for Precision Medicine (TOPMed) databases. Mutations were filtering by prediction softwares (Polyphen2, SIFT, MutationTaster). In the analysis we followed the below listed main steps: First, we set the following quality scores: GATK (QUAL) > 50, GQ > 40, RD > 4. We used the SureCall software to filter the variants. The missense, nonsense, indels and splice site variants were selected with a MAF (minor allele frequency) of < 0.01. All SNVs with higher score than three of SureCall were excluded. Next filtering was performed with in silico prediction software (Polyphen2, Mutation taster: FATHMM). The confirmation and segregation analyses of the alterations were performed by Sanger sequencing. Finally we used the ACMG (American College of Medical Genetics) guideline to indicate pathogenicity of the variants.

The 33-year-old female was a member of a non-consanguineous family. She had two healthy siblings, her father had multiple brain ischemia and died of esophageal cancer, her mother was healthy. The proband’s first symptom, progressive hypoacusis (sensorineural hearing loss) started at the age of 4. The progressive hypoacusis is currently very severe. The progressive gait disturbance started at age 12. Currently, she is wheelchair-bound due to the severe ataxia and associated lower limb weakness. She often complains of myalgia and muscle crumps. She has never had a period, the treatment with different endocrinological medications were not successful. She has few social relationships due to her querulous and paranoid personality. Neurological examination revealed bilateral horizontal (and also downbeat) nystagmus, severe hearing loss on both sides, moderate dysarthria, pes cavus on both sides, moderate atrophy, severe, predominantly proximal spastic paraparesis in the lower limbs, increased deep tendon reflexes, and bilateral pyramidal signs on both upper and lower limbs. She had severe truncal and limb ataxia, and upper limb dysdiadochokinesis. Slight distal type sensory deficit was detected as well in the lower limbs. Psychiatric examination observed moderate depression, anxiety, querulous behavior and paranoid personality traits. Her intellectual ability was intact. SCL-90-R detected higher scores on the hostility and somatization subscales.

**Fig. 1:**
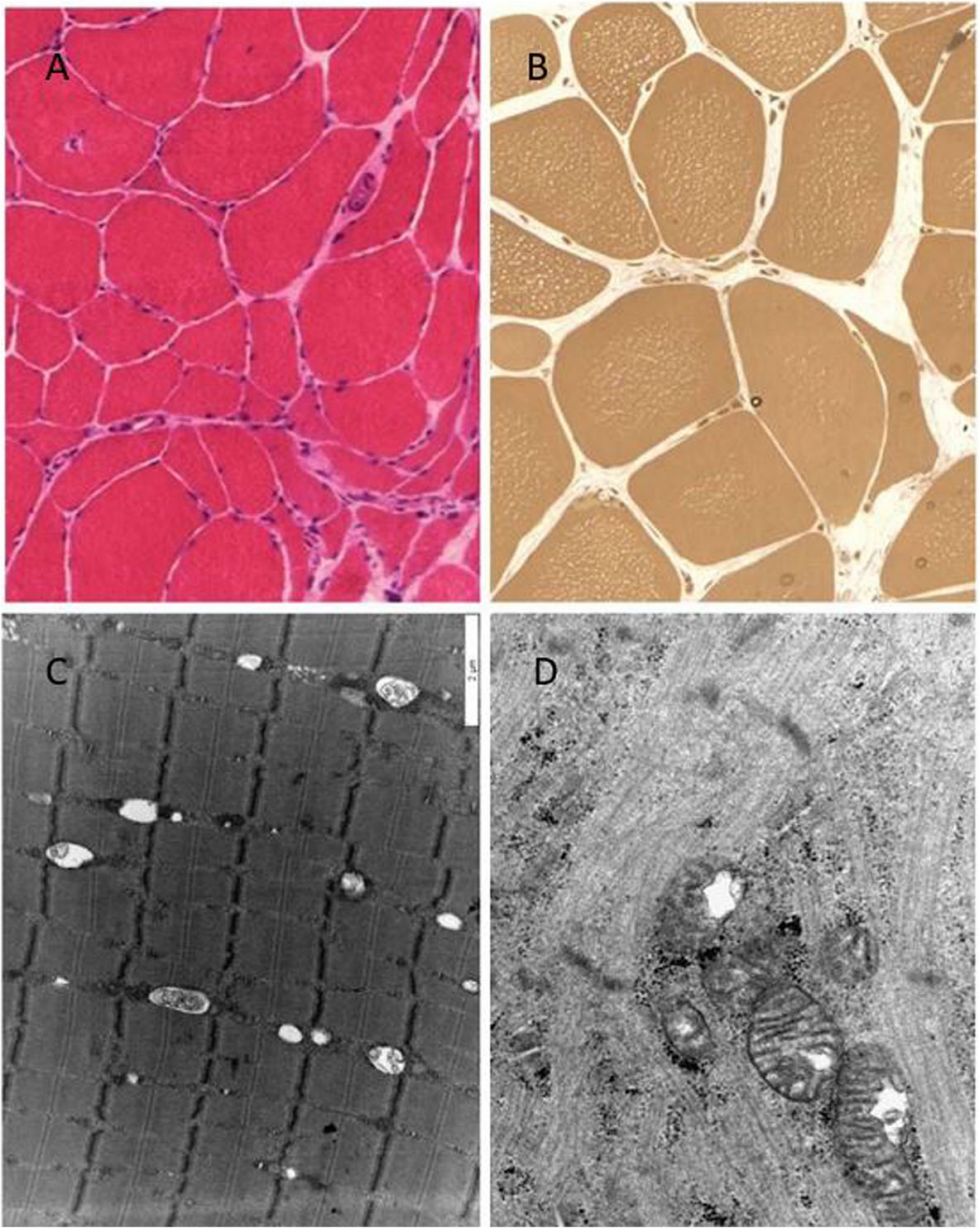
Neuropathological investigations. **a** Predominantly neurogenic alteration can be observed in the muscle tissue (large caliber variation, angular atrophic muscle fibers) H&E staining X250. **b** In the semithin section, many muscle fibers contained centrally numerous small vacuoles. Semi-thin section X350. **c** Electron microscopic picture of the muscle specimen. Lipid vacuoles are attached to the mitochondria. x **d** Enlarged mitochondria with abnormal cristae in the muscle tissue. × 24,000

**Fig. 2:**
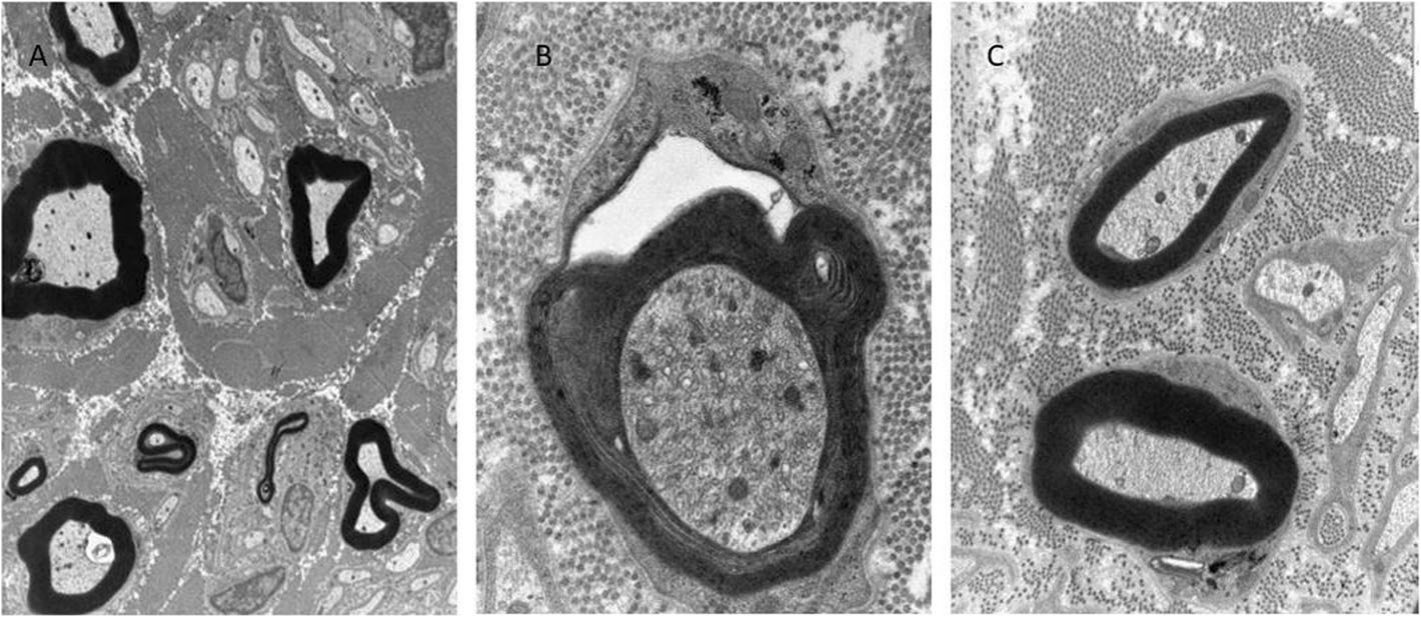
Electron microscopic pictures of the sural nerve biopsy specimen. **a** Complex group of degenerated and regenerated myelinated fibers. In one myelinated fiber, adaxonal vacuole is present, in another one, a myelin-like figure can be observed. × 8400. **b** Vesicular widening of the myelin in the region of a Schmidt-Lanterman cleft. In the Schwann cell and in the axon, slightly enlarged mitochondria (white arrows) are present. The myelin sheaths are slightly decompacted (dotted arrows). × 20.000 C. In a Schwann cell, a pi granula is present. × 16.000

The laboratory investigations detected her sex hormone levels (FSH, LH, estradiol) in the postmenopausal range, which is in line with primary amenorrhea: FSH 101.04 IU/L (norm: postmenopausal: 25.8–134.8 IU/L) LH: 49.22 IU/L (norm: postmenopausa:7.7–58.5 IU/L), estradiol 5 pg/ml (norm: postmenopausal: < 10–39.2 pg/mL), DHEA-S: 5.78 umol/L (norm: 1.65–11 umol/L). Prolactin, progesterone end total testosterone levels were normal. Elevated IGF-1 levels could be detected (1.65–11 umol/L, norm: 50–175 ng/mL). At age 33, her serum CK, LDH, and lactate levels were in the normal range, but previously (at age 28), laboratory analysis observed slightly elevated CK and lactate levels. EEG (electroencephalography) detected mild cortical dysfunction. ENG (electroneurography) observed severe mixed type neuropathy. The histological analysis of the muscle biopsy from tibial anterior muscle revealed signs of chronic neurogenic atrophy (Fig. 1a, b). Oxidative enzyme reaction could not detect any ragged red or blue fibers, COX staining was pale in the center of several muscle fibers, but COX-negative fibers were not present. Electron microscopy detected slightly enlarged mitochondria with abnormal cristae in the muscle (Fig. 1d). Next to the mitochondria, frequently lipid droplets were attached (Fig. 1c). The sural nerve biopsy found predominantly axonal type neuropathy. Complex group of degenerated and regenerated myelinated fibers were observed (Fig. 2a). In one myelinated fiber, adaxonal vacuole was present. Enlarged mitochondria were present in the axons and in the Schwann cells as well (Fig. 2b). In some axons, osmiophil globoids were seen, and in some Schwann cells, pi granules were present (Fig. 2c). In some fibers, slight signs of demyelinating components, such as focal myelin decompactation, were observed (Fig. 2b).

**Fig. 3:**
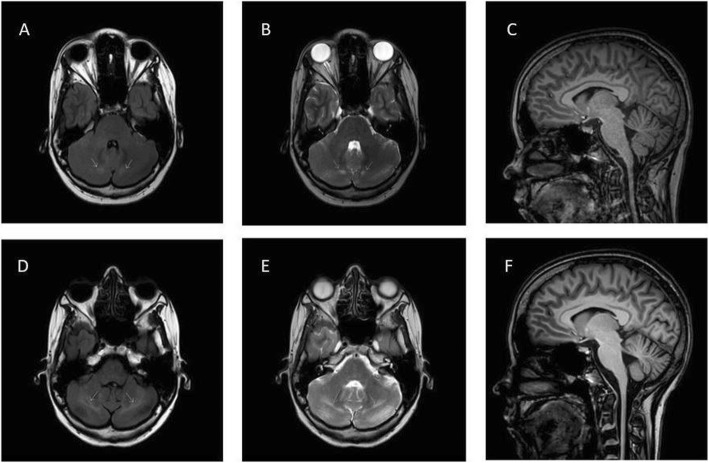
Brain MRI of the patient. **a** (FLAIR horizontal), **b** (T2 horizontal) and **c** (sagittal horizontal) are MRI examinations performed in 2008, while **d** (FLAIR horizontal), **e** (T2 horizontal) and **f** (sagittal) was performed in 2016. The hyperintense signs (white arrows) in the cerebellum are clearly visible on the images and show progression between the two examinations. Cerebellar, medulla oblongata, and cervical spinal cord atrophy can also be observed

The first MRI was performed at the age of 25. On the axial images, enlarged 4th ventricle and hyperintense signs in the cerebellum could be detected. Medulla oblongata, cervical spinal cord, and cerebellar atrophy were also present (Fig. 3a, b, c). The second MRI at the age of 33 showed the same alterations, and the progression of the hyperintense signs in cerebellum and the cerebellar atrophy that had been previously detected (Fig. 3d, e, f).

**Fig. 4:**
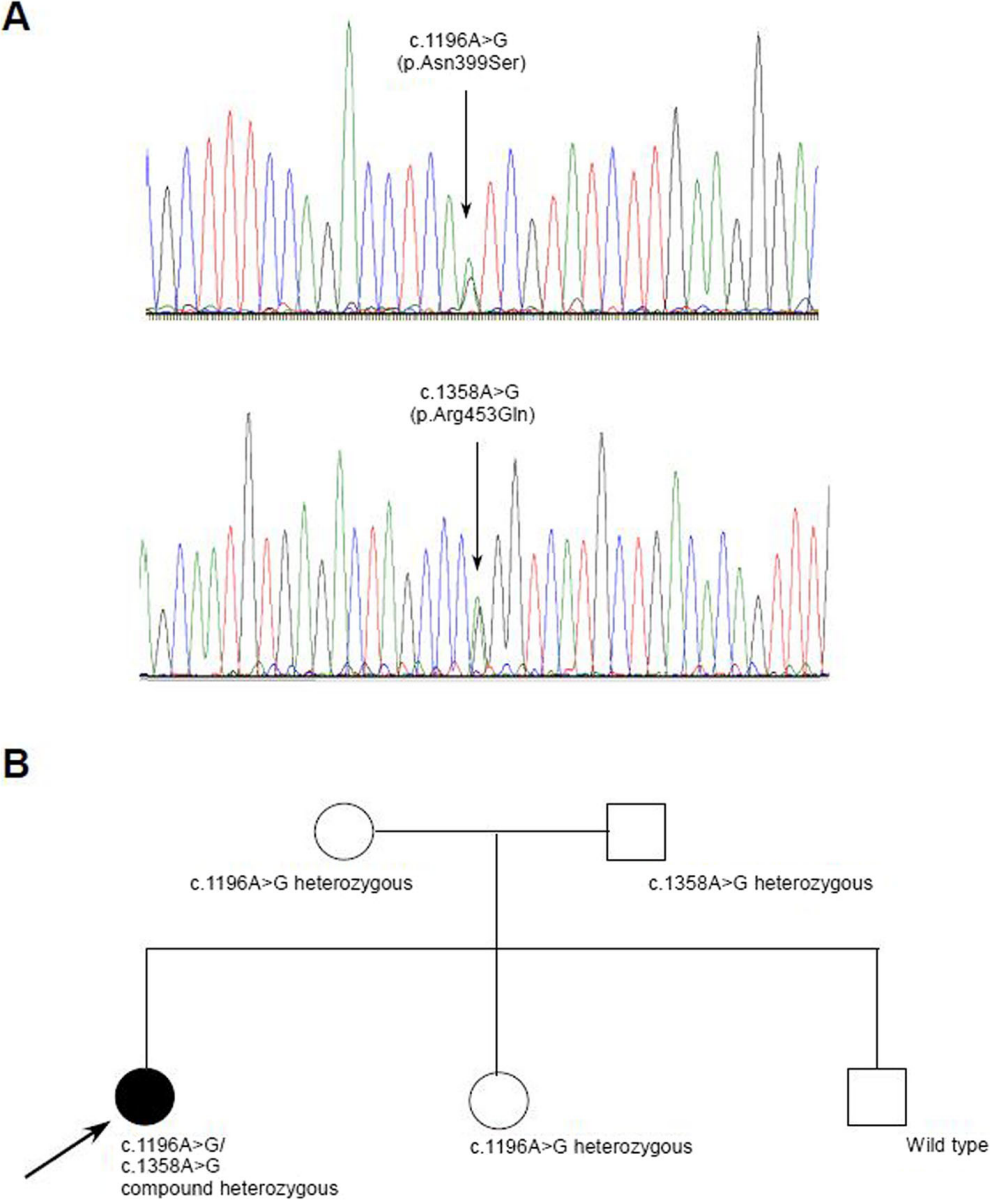
**a** Sanger traces of the identified mutations **b** pedigree information with segregation information. The mother is heterozygous for c.1196A > G, the father is heterozygous for c.1358A > G, and one female sibling is heterozygous for c.1196A > G, the other healthy sibling does not carry any of the examined mutations

The genetic investigation started with the establishment of the patients karyotype, which is 46XX. The investigation of the mitochondrial DNA hotspots detected multiple mtDNA deletions in the muscle tissue. The sequencing of the *POLG1, HSD17B4* and *HARS2* genes did not reveal any pathogenic alterations. An NGS (next generation sequencing) panel of 51 genes (Additional file 1: Table S1) responsible for the intergenomic communication revealed a compound heterozygous mutation (c.1196 A > G, (p.Asn399Ser)(rs863223921), c.1358 G > A, (Arg453Gln) in the *TWNK* gene (NM_021830.5). Prediction scores provided by Annovar [1] were the following: c.1196A > G: Polyphen2: possibly damaging, MutationTaster: disease-causing, FATHMM: tolerated, c.1358G > A: Polyphen2: benign, MutationTaster: disease causing, FATHMM: deleterious. Both rare variants could be confirmed in the proband via Sanger sequencing. The segregation analysis of the parents by Sanger sequencing revealed the c.1358G > A variation in the father, the c.1196 A > G in the mother in heterozygous form. (Fig. 4a, b) Copy number variations in the *TWNK* gene were not present. The variant frequency for both alterations are the following: the c.1196A > G (N399S) (rs863223921) mutation was found in the Genome Aggregation Database (genomeAD) in non-Finnish European with MAF: 3.978e-06 in the Trans-Omics for Precision Medicine (TOPMed) database with MAF: 0.00003, while this was not present in the 1000 Genomes Project Phase 3 (1000G), and Exome Aggregation Consortium (ExAc) databases. The c.1358 G > A (R453Q) (rs760988188) mutation was found in the gnomAD in non-Finnish European with MAF 8.79e-06, in the TOPMed with MAF: 0.00001, while this was not present in the 1000Gp3 and ExAc databases. Both the previously published variant the c.1196 A > G, and the novel c.1358 G > A variant described by us are likely pathogenic based on the ACMG guideline (both PM2, PP1, PP2, PP3, PP4).

One of her healthy siblings is carrying the c.1196A > G mutation in heterozygous form, her mother carries the same mutation in a heterozygous form and her father is carrying the c.1358A > G mutation in heterozygous form.

## Discussion and conclusion

Here, for the first time, we are reporting a comprehensive clinical and morphological (imaging and neuropathological) characterization of patient with *TWNK* (Twinkle) gene associated Perrault syndrome. Besides the classical signs of this syndrome, such as severe hypoacusis, ovarium dysgenesis and sensorimotor neuropathy, severe pyramidal tract lesion was observed as well, which was previously not reported in any of the cases. We are also the first to report the presence of cerebellar hyperintense lesion associated with Perrault syndrome. Until now, there have been only four previous reports describing mutations in *TWNK* gene alterations in association with Perrault syndrome. The patient reported in this study had compound heterozygous mutations in the *TWNK* gene. One of the disease-causing mutations, the c.1196A > G (Asn399Ser) variant was already associated with Perrault syndrome by Demain in a Caucasian family [3]. The second rare variant is a novel, likely pathogenic variant found by our group: c.1358G > A (Arg453Gln). There is one common clinical finding in all of the known *TWNK*-dependent Perrault syndrome patients’ ataxia and sensory neuropathy. Interestingly, PEO was not a clinical feature in our case, but it was present in three of the previously described cases. Spastic paraparesis and pyramidal signs seem to be unique in our patient. In the other cases, hyporeflexia or areflexia was present. Based on our observation, *TWNK*-associated Perrault syndrome could also be considered as a rare type of spastic ataxia. Intellectual abilities seemed intact in all reported cases, and psychiatric symptoms are described in Perrault syndrome, but not in the *TWNK*-associated form. In the case presented by us, ovarium agenesis and uterus hypoplasia were present with elevated LH and FSH and lower estrogen levels. In previous studies, gonadal dysgenesis, streak ovaries were mentioned with the indications of elevated LH and FSH levels with low estradiol level [3]. We also detected elevated IGF-1 levels. Dynamically changing serum CK and lactate levels were observed in our patient, while in previous studies, elevated CK level was observed in two related patients, and elevated lactate level was evaluated in one of two affected sisters.

In the previously published *TWNK*-associated Perrault patients, brain MRI found normal status in two cases, non-specific white matter changes in two patients, and cerebellar atrophy in other cases. In our patient, medulla oblongata and cervical spinal cord atrophy, moderate cortical atrophy, and hyperintense signs in the cerebellum were detected. Hyperintense signs in the cerebellum is an interesting novelty, it had not been described previously. The atrophy usually reported in Perrault syndrome typically affected the cerebellum, not the medulla oblongata and cervical spinal cord like in our case [3, 12, 14]. In two previous cases without MRI brain abnormalities, cerebral SPECT (single photon emission computed tomography) scan could identify cerebellar hypoperfusion [12]. We do not have information about EEG examinations in other *TWNK*-dependent Perrault syndrome cases, but upon examination, we could detect mild cortical dysfunction. The presence of the mixed type sensory neuropathy is in line with the previous findings. In both the muscle and the sural nerve tissue, slight mitochondrial abnormalities were present.

Perrault syndrome is still not fully characterized genetically, and, in several cases, the genetic diagnosis is missing. By our report, we could add a novel mutation (c.1358G > A) to the spectrum and confirm a recently described disease-causing variation (c.1196A > G). In the future, it will be necessary to continue to identify new mutations, to find new patients, and to follow-up the existing patients to better characterize the phenotype and be able to provide the best possible care by understanding the disease.

Our observation confirms the reports that Twinkle protein dysfunction is associated with Perrault syndrome. Our results demonstrate that *TWNK*-associated Perrault syndrome has a much broader phenotype than originally was published. The coexistence of severe hypoacusis, spastic limb weakness, ataxia, polyneuropathy, gonadal dysgenesis, and hyperintense signals in the cerebellum, and the presence of the mtDNA multiple deletion could indicate the impairment of the *TWNK* gene. This is the first report about pyramidal tract involvement and cerebellar hyperintense MRI signs associated to *TWNK*-related Perrault syndrome. Our observations confirm the pathogenicity of the c.1196A > G mutation and identify a new pathogenic mutation as c.1358G > A.

## Supplementary information

**Additional file 1: Table S1.** List of the genes used in the NGS panel.

## Data Availability

The datasets used and/or analyzed during the current study are available from the corresponding author on reasonable request. The datasets generated and analysed during the current study are available in the NEPSYBANK repository at the following website: http://semmelweis.hu/genomikai-medicina/nepsybank-repository/ For further information reference code is the title of the article.

## Abbreviations

1000G: 1000 Genomes
ACMG: American College of Medical Genetics
CK: Creatine kinase
CNS: Central nervous system
COX: Cytochrome C oxydase
EEG: Electroencephalography
ENG: Electroneurography
ExAC: Exome Aggregation Consortium
GenomeAD: Genome Aggregation Database
GSI: Global severity index
MAF: Minor allele frequency
NCBI: National Center for Biotechnology Information
NCS: Nerve conduction studies
NGS: Next generation sequencing
PCR: Polymerase chain reaction
PEO3: Progressive external ophthalmoplegia 3
SAS: Statistical Analysis System
SCA: Spinocerebellar ataxia
SCL-90-R: Symptom Checklist-90-Revised
SPECT: Single photon emission computed tomography
TOPMed: Trans-Omics for Precision Medicine
