# Supplementary figures and images for: Broadening the phenotype of the TWNK gene associated Perrault syndrome
Source: BMC Med Genet. 2019 Dec 18;20:198. doi: 10.1186/s12881-019-0934-4 (PMC6921552; doi:10.1186/s12881-019-0934-4)

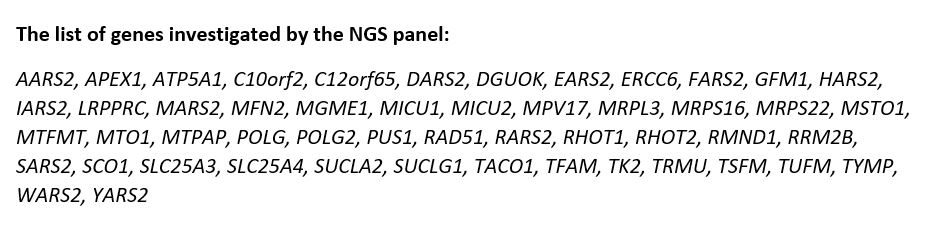

Supplement: Supplementary file 1 — Additional file 1: Table S1. List of the genes used in the NGS panel. [file 12881_2019_934_MOESM1_ESM.jpg]
